# The Integration of Metabolomics and Transcriptomics Provides New Insights for the Identification of Genes Key to Auxin Synthesis at Different Growth Stages of Maize

**DOI:** 10.3390/ijms232113195

**Published:** 2022-10-30

**Authors:** Zhenzhong Jiang, Honglin Zhang, Peng Jiao, Xiaotong Wei, Siyan Liu, Shuyan Guan, Yiyong Ma

**Affiliations:** 1 College of Life Sciences, Jilin Agricultural University, Changchun 130118, China; 2 Joint International Research Laboratory of Modern Agricultural Technology, Ministry of Education, Changchun 130118, China; 3 College of Agronomy, Jilin Agricultural University, Changchun 130118, China

**Keywords:** metabolomics, transcriptomics, auxin synthesis, growth stages, maize

## Abstract

As a staple food crop, maize is widely cultivated worldwide. Sex differentiation and kernel development are regulated by auxin, but the mechanism regulating its synthesis remains unclear. This study explored the influence of the growth stage of maize on the secondary metabolite accumulation and gene expression associated with auxin synthesis. Transcriptomics and metabonomics were used to investigate the changes in secondary metabolite accumulation and gene expression in maize leaves at the jointing, tasseling, and pollen-release stages of plant growth. In total, 1221 differentially accumulated metabolites (DAMs) and 4843 differentially expressed genes (DEGs) were screened. KEGG pathway enrichment analyses of the DEGs and DAMs revealed that plant hormone signal transduction, tryptophan metabolism, and phenylpropanoid biosynthesis were highly enriched. We summarized the key genes and regulatory effects of the tryptophan-dependent auxin biosynthesis pathways, giving new insights into this type of biosynthesis. Potential MSTRG.11063 and MSTRG.35270 and MSTRG.21978 genes in auxin synthesis pathways were obtained. A weighted gene co-expression network analysis identified five candidate genes, namely *TSB* (Zm00001d046676 and Zm00001d049610), *IGS* (Zm00001d020008), *AUX2* (Zm00001d006283), *TAR* (Zm00001d039691), and *YUC* (Zm00001d025005 and Zm00001d008255), which were important in the biosynthesis of both tryptophan and auxin. This study provides new insights for understanding the regulatory mechanism of auxin synthesis in maize.

## 1. Introduction

Maize (*Zea mays*) is an annual herb in the Gramineae family that is native to Central and South America. Maize is cultivated in tropical and temperate regions and throughout China. It is a staple food crop, and a crucial source of feed and industrial raw materials. The growth and development of maize plants has been categorized into a number of vegetative and reproductive stages [1], during which diverse phytohormones exert important regulatory effects [2].

As is typical for angiosperms, maize synthesizes numerous endogenous hormones, such as auxin, gibberellin, abscisic acid, and zeatin. Auxin plays an important role in regulating the growth and development of stems, buds, and roots [3]. The compounds indole-3-acetic acid (IAA), 1H-indole-3-butanoic acid (IBA), 1-naphthalene-acetic acid (NAA), 2-naphthoxy-acetic acid (BNOA), 2,4-dichlorophenoxyacetic acid (2,4-D), 4-chloro-phenoxyacetic acid (4-CP), and 4-iodophenoxyacetic acid (4-iodo pyridine) are collectively termed auxins [4]. Indoleacetic acid was the first plant hormone to be discovered and is mainly distributed in actively growing tissues, such as stem meristems, leaf primordia, young leaves, developing fruit and seeds, and pollen grains [5]. Auxins promote the formation of lateral roots and adventitious roots, adjust flowering and sex differentiation, regulate fruit set and development, and control apical dominance [6,7]. Most of the essential genes associated with auxin synthesis are involved in tryptophan-dependent biosynthesis, whereas the tryptophan-independent pathway is poorly understood because the essential genes involved have not yet been cloned [8]. Based on the main intermediate products of IAA synthesis, tryptophan-dependent biosynthesis is usually divided into four pathways: the indole-3-acetaldoxime (IAOx) pathway, tryptamine pathway, indole-3-acetamide (IAM) pathway, and indole-3-pyruvic acid (IPA) pathway [9]. With the clarification of the key catalytic enzyme functions in the auxin synthesis pathways, some authors have divided tryptophan-dependent auxin synthesis into the CYP79B pathway, YUCCA (YUC) pathway, indoleacetamide pathway, and indolylpyruvate pathway [10]. The CYP79B protein family in the IAOx pathway converts tryptophan into indole-3-acetaldoxime, but the conversion of indole-3-acetaldoxime to IAA remains unclear [11]. Tryptamines in the tryptamine pathway have long been considered to be precursors of IAA synthesis. In the tobacco (*Nicotiana tabacum*) apical meristem and callus, and shoots of *Solanum tuberosum* and *Hordeum vulgare*, isotope-labeled 14C tryptamines are converted to IAA [12]. Phelps and Sequeira (1967) [13] identified a tryptamine auxin synthesis pathway in tobacco. The indoleacetamide pathway has been detected in various microorganisms. Endogenous indole-3-acetamide has been detected in *Citrus reticulata*, *Prunus jamasakura*, and *Cucurbita maxima*. However, for a long time, researchers generally believed that the indoleacetamide pathway did not exist in plants. With the improvement of analytical procedures and detection equipment, endogenous indole-3-acetamide has been accurately detected in *Arabidopsis thaliana*, maize, rice (*Oryza sativa*), and tobacco [14,15].

The IPA pathway is an important pathway for auxin synthesis in microorganisms [16]. Gene functional analysis, in vitro experiments, and isotope tracing have shown that the *YUC* gene family catalyzes the direct conversion of indolylpyruvate to IAA [17,18]. To date, *YUC* homologous genes have been cloned in diverse plant species, such as *Petunia hybrida*, tomato (*Solanum lycopersicum*), *Ipomoea nil*, and rice, indicating that *YUC* genes exert similar effects on the growth and development of diverse plant species [19]. Therefore, the IPA pathway is considered to be the most fundamental, primary auxin synthesis pathway so far elucidated in plants [20]. Although several auxin synthesis pathways have been identified in plants, further study is required to clarify the regulatory mechanism of each pathway.

The jointing, tasseling, and pollen-release stages of maize growth are associated with the development of the sexual organs. Auxins play an essential role in this process. The present study utilized two inbred maize lines with different growth cycles, and compared the expression of auxin synthesis genes and the accumulation of metabolites in the same line between the jointing and tasseling, and tasseling and pollen-release stages, and between two lines at the same growth stage. The aim was to identify the auxin synthesis pathway genes that exert pivotal effects on the growth and development of maize, and to characterize the spatiotemporal expression patterns of these specific genes. The transcriptomes of two inbred maize lines with short and long growth periods were analyzed to explore the differential gene expression associated with the auxin biosynthesis pathway in leaf tissues. In addition, the metabonomes were compared to detect differential metabolites in the leaves at different growth stages. Integration of the metabonome and transcriptome data enabled screening for the key candidate genes and metabolites of the auxin biosynthesis pathways. The present results provide novel insights into auxin biosynthesis in maize.

## 2. Results

### 2.1. Determination of Trp and IAA Contents and Identification of DEGs

**Figure 1:**
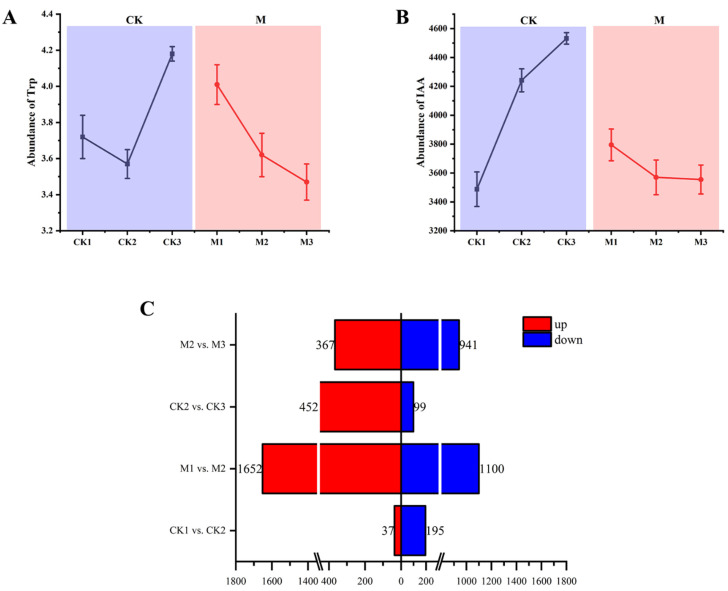
Total indole−3−acetic acid (IAA) and tryptophan contents in the leaves of maize and differentially expressed genes (DEGs) in different comparison groups. (**A**) Tryptophan content and (**B**) IAA content in leaves of maize at different growth stages. (**C**) Number of DEGs in the different comparison groups.

Certain differences between the changes in tryptophan and IAA contents in the leaves at different growth stages were observed. The Trp content initially decreased and then increased from CK1 to CK3, whereas it decreased consistently from M1 to M3 (Figure 1A). The IAA content showed an upward trend from CK1 to CK3, which was the opposite of the change observed from M1 to M3 (Figure 1B). The trend for the Trp content in the CK1–CK2 stages was opposite to that of IAA content, whereas the trend for the Trp content in the CK2–CK3 stages was identical to that of IAA content. In contrast, the changes in Trp content from M1 to M3 were identical to the changes in IAA content.

Eighteen cDNA libraries were constructed according to the sampling time: jointing stage, CK1 (CK1217-1, CK1217-2, and CK1217-3) and M1 (M1217-1, M1217-2, and M1217-3); tasseling stage, CK2 (CK1222-1, CK1222-2, and CK1222-3) and M2 (M1222-1, M1222-2, and M1222-3); and pollen-release stage, CK3 (CK1231-1, CK1231-2, and CK1231-3) and M3 (M1231-1, M1231-2, and M1231-3). For example, CK1-1 means the first sample of CK at jointing stage(CK1217-1). Transcriptome sequencing screening yielded 15,347,554,632 raw reads, resulting in 152,913,838 high-quality clean reads after performing quality control. The reads ranged from 103 to 20,570 bp in length.

We constructed four subgroups to analyze the transcriptome data for the CK and M lines at different growth stages: CK1 vs. CK2, CK2 vs. CK3, M1 vs. M2, and M2 vs. M3. In total, 4843 DEGs were screened with the criterion *p* < 0.05. The number of upregulated and downregulated DEGs from each control group is shown in Figure 1C. The M1 vs. M2 group had the largest number of DEGs, comprising 1652 upregulated DEGs and 1100 downregulated DEGs. The number of DEGs in the CK1 vs. CK2 group was 232, of which 37 were upregulated and 195 were downregulated. A significant difference in the DEGs between the M1 vs. M2 and CK1 vs. CK2 groups was observed. The number of DEGs in the CK2 vs. CK3 group was 551, and that of the M2 vs. M3 group was 1038; a significant difference in the number of DEGs between these two groups was also observed.

### 2.2. GO Enrichment Analysis of DEGs

**Figure 2:**
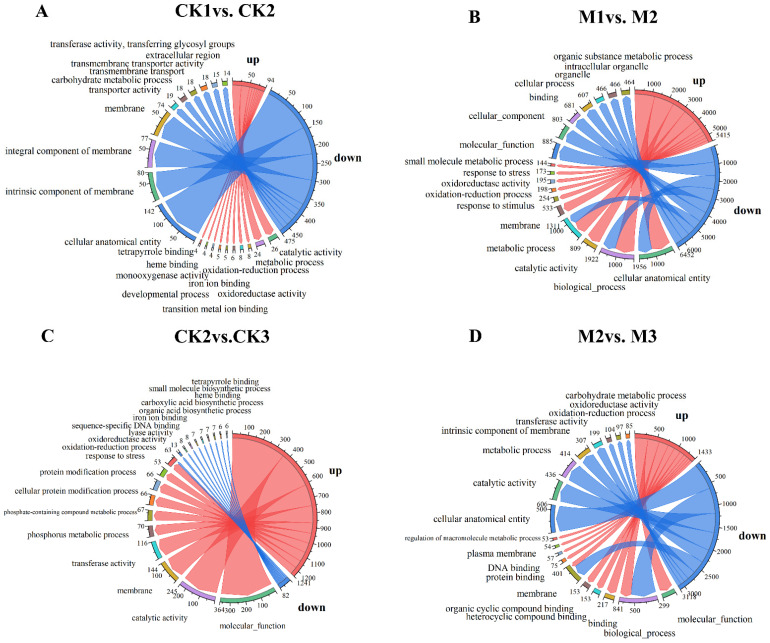
Circos plot of gene ontology (GO) enrichment of differentially expressed genes (DEGs). The right half-circle indicates the number of up- and down-regulated DEGs in each comparison group; red indicates up-regulation and blue indicates down-regulation. The outermost circle of the left half-circle indicates the GO terms and different colors represent different GO terms; the inner circle indicates the number of up- and down-regulated DEGs for each GO term. Arrows point from up- and down-regulated genes to GO terms, and arrow thickness indicates the abundance of up- and down-regulated genes for each GO term. (**A**) CK1 vs. CK2, (**B**) M1 vs. M2, (**C**) CK2 vs. CK3, and (**D**) M2 vs. M3.

The 4843 DEGs screened from all samples were enriched in 1824 GO terms, among which the biological process category accounted for the highest proportion (61.23%), followed by molecular function (28.44%) and cellular component (10.33%). The function and degree of enrichment between each group (i.e., CK1 vs. CK2, M1 vs. M2, CK2 vs. CK3, and M2 vs. M3) differed significantly (Figure 2). Among the 10 most highly enriched GO terms in each group, five terms were enriched in most groups, including membrane (GO:0016020), oxidation reduction process (GO:0055114), catalytic activity (GO:0003824), cellular analytical entity (GO:0110165), and biological process (GO:0008150). A significant difference in the number of enriched terms was observed between CK1 vs. CK2 and M1 vs. M2 (Figure 2A,B). The number of enriched terms was highest in M1 vs. M2-up (Figure 2B), with 1165 genes enriched in cellular anatomical entity (GO:0110165) and 1162 genes enriched in biological process (GO:0008150). Finally, we collected key genes in the pathway for hormone synthesis and tryptophan synthesis enrichment, where the differential genes involved in auxin synthesis include Zm00001d025005, Zm00001d004467, and Zm00001d042809 MSTRG.11063, and the differential genes related to tryptophan synthesis include Zm000001d049610 and Zm00001d020008. Unrepresented genes were found in these differential genes, and these unrepresented genes may be potential genes during auxin synthesis. The expression level of the MSTRG.11063 gene in M is higher than that of CK. Changes in the expression of these differential genes lead to a decrease in the content of auxin synthesized by M.

In addition, significant differences in the genes and enrichment functions were observed between the paired groups CK1 vs. CK2 + M1 vs. M2 and CK2 vs. CK3 + M2 vs. M3. The CK1 vs. CK2 and M1 vs. M2 (Figure 2A,B) groups were mainly enriched in cellular anatomical entity (GO:0110165) and biological process (GO:0008150), with the highest proportion of enriched terms in the biological process category and the lowest in the cellular component category (Figure 2A,B). By contrast, CK2 vs. CK3 and M2 vs. M3 were mainly enriched in membrane (GO:0016020) and oxidation reduction process (GO:0055114); the highest proportion of enriched terms was in the biological process category and the lowest proportion was in the cellular component category (Figure 2C,D). Thus, the terms “biological process” and “metabolic process” were significantly enriched in the biological process category in all four groups. “Catalytic activity” and “molecular function” were significantly enriched in the molecular function category. With regard to cell formation, the most highly enriched terms were “cellular anatomical entity,” “cellular component,” and “intrinsic component of membrane”.

### 2.3. KEGG Pathway Enrichment Analysis of DEGs

**Figure 3:**
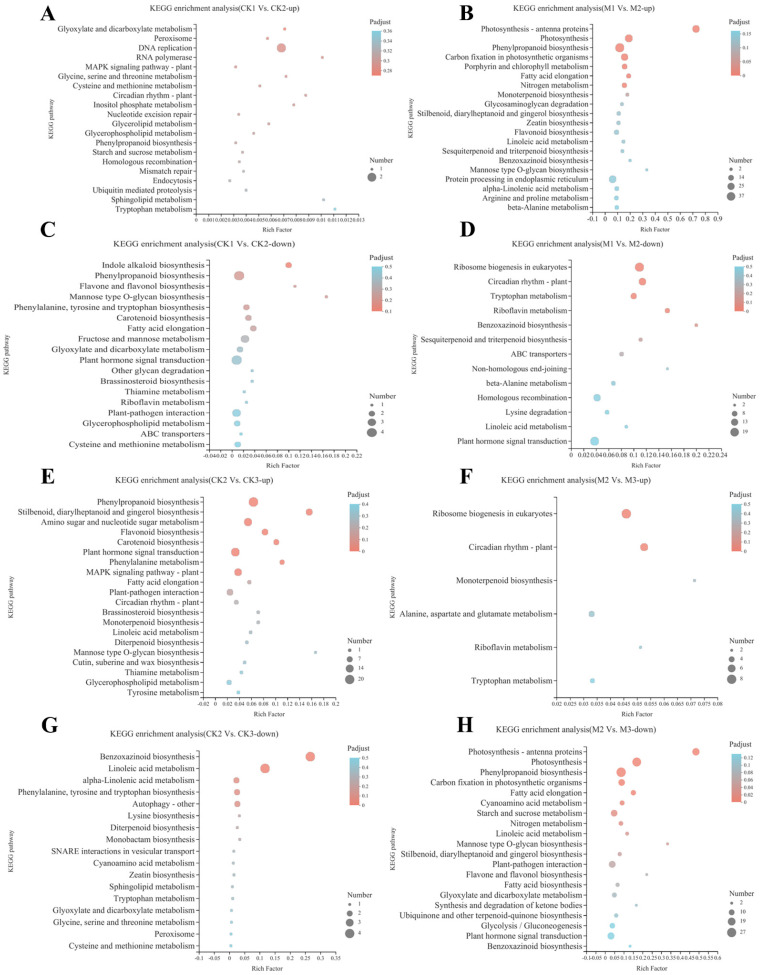
Bubble diagram of KEGG pathway enrichment of differentially expressed genes. The *Y*−axis shows the name of the pathway and the *X*−axis represents the Rich factor. The larger the Rich factor, the greater the degree of enrichment. (**A**) M1 vs. M2−up, (**B**) CK1 vs. CK2−up, (**C**) M2 vs. M3−up, (**D**) CK2 vs. CK3−up, (**E**) M1 vs. M2−down, (**F**) CK1 vs. CK2−down, (**G**) M2 vs. M3−down, and (**H**) CK2 vs. CK3−down.

The 4843 DEGs were mapped to 205 KEGG pathways. The highest number of DEGs were enriched in metabolism, whereas the fewest DEGs were enriched in cellular processes. The upregulated DEGs of the CK1 vs. CK2 group were mainly enriched in DNA replication (map00940), whereas the downregulated DEGs were mainly enriched in phenylpropanoid biosynthesis (map00940), plant hormone signal transduction (map04075), fructose and mannose metabolism (map00051), and plant–pathogen interaction (map04626) (Figure 3A). The upregulated DEGs of the M1 vs. M2 (Figure 3B) group were mainly enriched in phenylpropanoid biosynthesis (map00940), photosynthesis (map00195), protein processing in endoplasmic reticulum (map04141), and photosynthesis–antenna proteins (map00196), whereas the downregulated DEGs were mainly enriched in ribosome biogenesis in eukaryotes (map03008), plant hormone signal transduction (map04075), circadian rhythm–plant (map04712), and homologous recombination (map03440). Plant hormone signal transduction (map04075) was mainly enriched in the upregulated DEGs of CK2 vs. CK3 and the downregulated DEGs of M2 vs. M3 (Figure 3E,H). The phenylpropanoid biosynthesis (map00940) and photosynthesis (map00195) pathways were mainly enriched among M2 vs. M3 downregulated DEGs (Figure 3H), whereas the benzoxazinoid biosynthesis (map00402) and linoleic acid metabolism (map00591) pathways were mainly enriched among CK2 vs. CK3 downregulated DEGs (Figure 3G). Subsequently, we screened for genes related to auxin and tryptophan synthesis in Plant hormone signal transduction (map04075), Indole alkaloid biosynthesis (map00901), Tryptophan metabolism (map00380), and included Zm000001d008255, Zm000001d034118, MSTRG.21978, MSTRG.31895, MSTRG.9004, MSTRG.31080, MSTRG.35990, MSTRG.5662, and MSTRG.17742. Zm0000001d008255 was expressed significantly more in M1 vs. M2. Zm000001d046676 and Zm00001d049610 were associated with tryptophan synthesis. Uncharacterized genes in auxin and tryptophan synthesis were also found.

In general, the enriched KEGG pathways in each group were mainly concentrated in amino acid metabolism and photosynthesis. However, although hormone signal transduction was not primarily enriched, its relevant genes were widely regulated in all groups (Figure 3B,C,E,G,H). Hormone signal transduction included plant hormone signal transduction (map04075), zeatin biosynthesis (map00908), and indole alkaloid biosynthesis (map00901). Amino acid metabolism included tryptophan metabolism (map00380), glycine, serine and threonine metabolism (map00260), and phenylalanine, tyrosine, and tryptophan biosynthesis (map00400). Photosynthesis mainly included photosynthesis (map00195) and photosynthesis–antigen proteins (map00196). The plant hormone signal transduction (map04075), indole alkaloid biosynthesis (map00901), and tryptophan metabolism (map00380) pathways are all involved in auxin synthesis and signal transduction.

### 2.4. Widely Targeted Metabolomics Analysis and Overall Metabolite Identification

**Figure 4:**
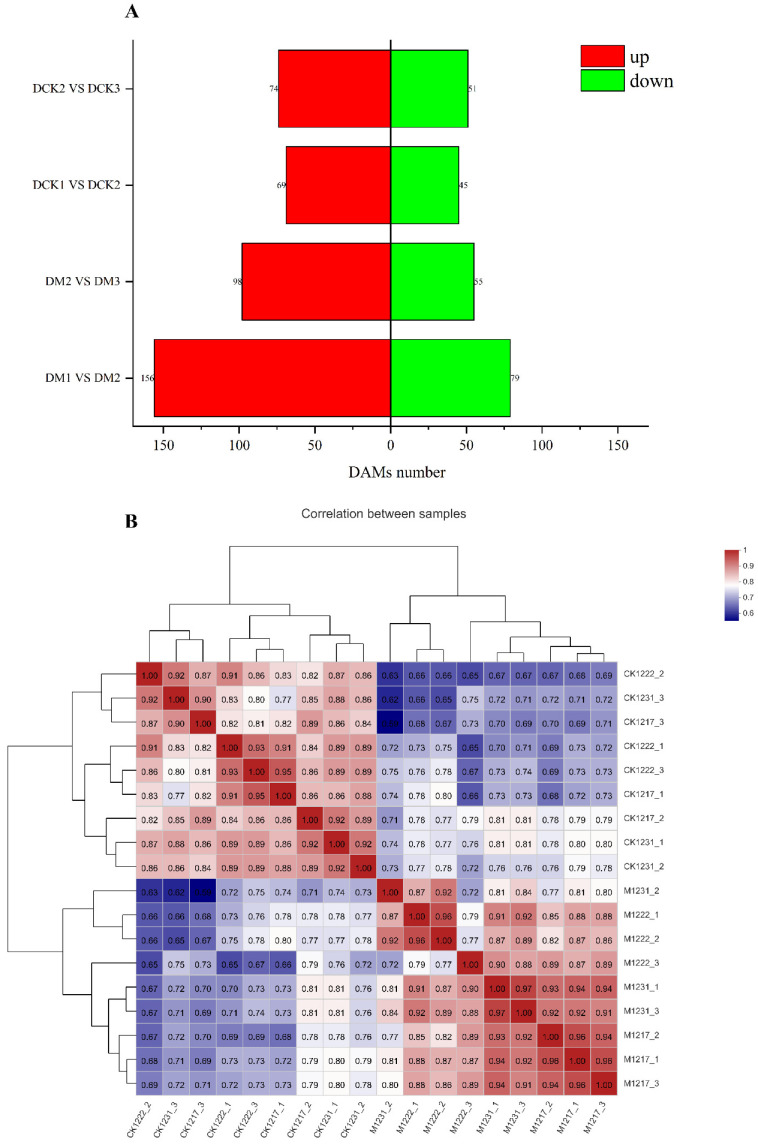
Metabolomic analysis of maize of different growth stages. (**A**) Distribution of up- and down-regulated DAMs. red is up, green is down. (**B**) Cluster heatmaps of the three biological replicates, red indicates a strong correlation, blue indicates a weak correlation.

A metabonomic analysis was conducted to explore the changes in metabolites in the leaves at different growth stages. A total of 1221 metabolites were screened, comprising mainly prenol lipids (132), organoxygen compounds (129), fatty acyls (113), carboxylic acids and derivatives (80), flavonoids (60), steroids and their derivatives (58), glycosphospholipids (42), benzone and substituted derivatives (34), coumarins and derivatives (26), phenols (23), cinnamic acids and their derivatives (22), and indoles and their derivatives (15). Cluster heatmaps (Figure 4B) of the three biological replicates of each sample revealed that each comparison group showed a high degree of similarity, indicating the strong reliability of the metabonomic data. To examine the trends in metabolic changes at the different growth stages, 297 metabolites were clustered using the K-means clustering method. Based on the accumulation patterns of the different metabolites, eight clusters were obtained (Figure S1A–H). We separately performed enrichment analyses on CK1 vs. CK2, CK2 vs. CK3, M1 vs. M2, and M2 vs. M3. As shown in Figure S2, the CK1 vs. CK2 group was most highly enriched in tryptophan metabolism (map00380), steroid biosynthesis (map00100), flavonoid biosynthesis (map00941), purine metabolism (map00230), and ABC transporters (map02010). The CK2 vs. CK3 group was highly enriched in flavonoid biosynthesis (map00941), phenylalanine metabolism (map00360), tryptophan metabolism (map00380), tropane, piperidine and pyridine alkaloid biosynthesis (map00960), and ABC transporters (map02010). The M1 vs. M2 group was primarily enriched in glycerophospholipid metabolism (map00564), beta-alanine metabolism (map00410), and tryptophan metabolism (map00380). The M2 vs. M3 group was mainly enriched in ABC transporters (map02010), arginine biosynthesis (map00220), and tryptophan metabolism (map00380). As shown in Figure S3, we analyzed a total of 12 metabolites in the tryptophan metabolic pathway, of which 7 were similar in structure to IPA, including Kynurenine, 2-Formylaminobenzaldehyde, Quinoline-4, 8-diol, 3-Methyldioxyindole, 5-Hydroxyindoleacetaldehyde, Kynurenic acid, and 5- Hydroxy-L-tryptophan; 1 metabolite was similar in structure to IAM, Indoleacetaldehyde; and 1 metabolite was similar in structure to TAM, 3-Methylindole, which is a potential substance in the auxin synthesis pathway. The highest number of potential metabolites being found in the IPA pathway suggests that the IPA pathway plays an important role in maize synthetic auxin and has higher synthetic diversity.

In addition, the changes in DAMs at the different growth stages were analyzed (Figure 4A). Accordingly, 627 DAMs were identified. Among the four groups, M1 vs. M2 included the most detected DAMs (235), while the fewest DAMs were detected in the CK1 vs. CK2 group (114). The number of upregulated and downregulated DAMs in each group is shown in Figure 4A. The groups M1 vs. M2 and M2 vs. M3 included more upregulated and downregulated DAMs than those of CK1 vs. CK2 and CK2 vs. CK3. With regard to unique and shared metabolites (Figure S1I), 23 metabolites were common to the four groups and mainly comprised phenylpropanoid biosynthesis (map00940), plant hormone signal transduction (map04075), flavonoid biosynthesis (map00941), ABC transporters (map02010), and glycerolipid metabolism (map00561). The CK1 vs. Ck2, CK2 vs. CK3, and M1 vs. M2 groups shared 51 DAMs, including glycerolipid metabolism (map00561), flavonoid biosynthesis (map00941), plant hormone signal transduction (map04075), cyanoamino acid metabolism (map00460), and tryptophan metabolism (map00380). The CK1 vs. CK2, CK2 vs. CK3, and M2 vs. M3 groups (Figure S1I) shared four DAMs, comprising phenylpropanoid biosynthesis (map00940), plant hormone signal transduction (map04075), glycerophospholipid metabolism (map00564), and carbon fixation in photosynthetic organisms (map00710). Among the plant hormone signal transductions are Trans-Zeatin (metab_8938), 3-Indoleacetic Acid (metab_8953), and jasmonic acid (metab_12197).

### 2.5. Integration of Transcriptomic and Metabonomic Analyses

**Figure 5:**
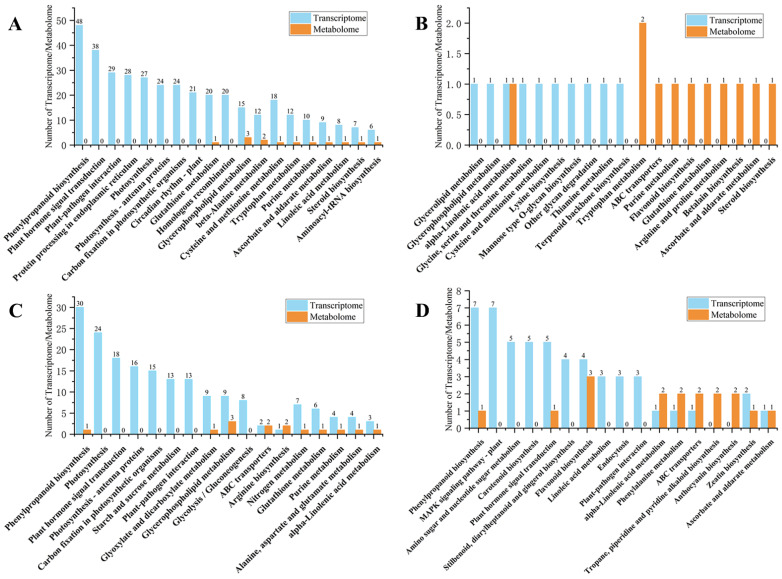
Mapping of differentially expressed genes (DEGs) and differentially accumulated metabolites (DAMs) to KEGG pathways. The *Y*-axis represents the number of transcriptomics (DEGs)and metabonomics(DAMs), and the *X*-axis represents the pathway. Blue is Transcriptom, orange is Medabolome. (**A**) CK1 vs. CK2, (**B**) M1 vs. M2, (**C**) CK2 vs. CK3, and (**D**) M2 vs. M3.

By integrating transcriptomic and metabonomic analyses, an improved understanding of the genetic regulation of metabolic processes can be gained. In this study, the DEGs and DAMs in each control group were mapped to KEGG pathways and the 10 most highly enriched pathways were obtained (Figure 5). For the CK1 vs. CK2 group, 376 DEGs and 13 DAMs were mapped to 19 KEGG pathways (Figure 5A). For the M1 vs. M2 group, 10 DEGs and 11 DAMs were mapped to 19 KEGG pathways (Figure 5B), of which phenylpropanoid biosynthesis (map00940) and plant hormone signal transduction (map04075) were the leading enriched pathways. For the CK2 vs. CK3 group, 182 DEGs and 14 DAMs were mapped to 19 KEGG pathways (Figure 5C), of which the five most enriched pathways were phenylpropanoid biosynthesis (map00940), MAPK signaling pathway–plant (map04016), amino sugar and nucleotide sugar metabolism (map00520), carotenoid biosynthesis (map00906), and plant hormone signal transduction (map04075). Finally, for the M2 vs. M3 group, 52 DEGs and 17 DAMs were mapped to 19 KEGG pathways, including phenylpropanoid biosynthesis (map00940), photosynthesis (map00195), and plant hormone signal transduction (map04075) (Figure 5D). A global KEGG enrichment analysis was performed on all metabolome and transcriptome data, as shown in Figure S4. This analysis revealed that the enriched pathways mainly included phenylpropanoid biosynthesis (map00940), plant hormone signal transduction (map04075), glycerolipid metabolism (map00561), glycerophospholipid metabolism (map00564), flavone and flavonol biosynthesis (map00944), MAPK signaling pathway—plant (map04016), phenylalanine, tyrosine and tryptophan biosynthesis (map00400), and tryptophan metabolism (map00380). Subsequently, we will perform an evolutionary analysis (Figure S5) of plant hormone signal transduction, indole alkaloid biosynthesis, auxin synthesis known genes, and uncharacterized genes in the tryptophan metabolism pathway. We can see that the MSTRG.11063 gene and the known gene *ZmYUCCA4* have a strong evolutionary relationship, and the MSTRG.35270 gene and the known gene *AUX2* have a strong evolutionary relationship. The MSTRG.21978 gene and the known gene *ZmCYP79A2* have a strong evolutionary relationship, and the remaining CYP uncharacterized genes have a distant evolutionary relationship with *ZmCYP79A2*, a potential gene in the growth synthesis pathway, which can provide a reference for the detection of auxin and tryptophan synthesis pathways.

The pathways that were significantly enriched among the top 10 pathways for the CK1 vs. CK2, CK2 vs. CK3, and M2 vs. M3 (Figure 5A,C,D) groups included phenylpropanoid biosynthesis (map00940) and plant hormone signal transduction (map04075). Among the groups CK1 vs. CK2, CK2 vs. CK3, and M2 vs. M3, three groups of 610 DEGs were enriched in plant hormone signal transduction (map04075). The DEGs of auxin signal transduction included Zm00001d039691, Zm00001d037674, Zm00001d018098, Zm00001d025005, Zm00001d004467, Zm00001d019527, Zm00001d008255, Zm00001d005439, Zm00001d008700, Zm00001d006283, Zm00001d051754, and Zm00001d044339. Thus, plant hormone signal transduction (map04075) in the stages of CK1 vs. CK2, CK2 vs. CK3, and M2 vs. M3 was indicated to play an important role, especially in auxin-related signal transduction.

### 2.6. Analysis of Auxin Biosynthesis-Related Metabolites and Gene Expression

**Figure 6:**
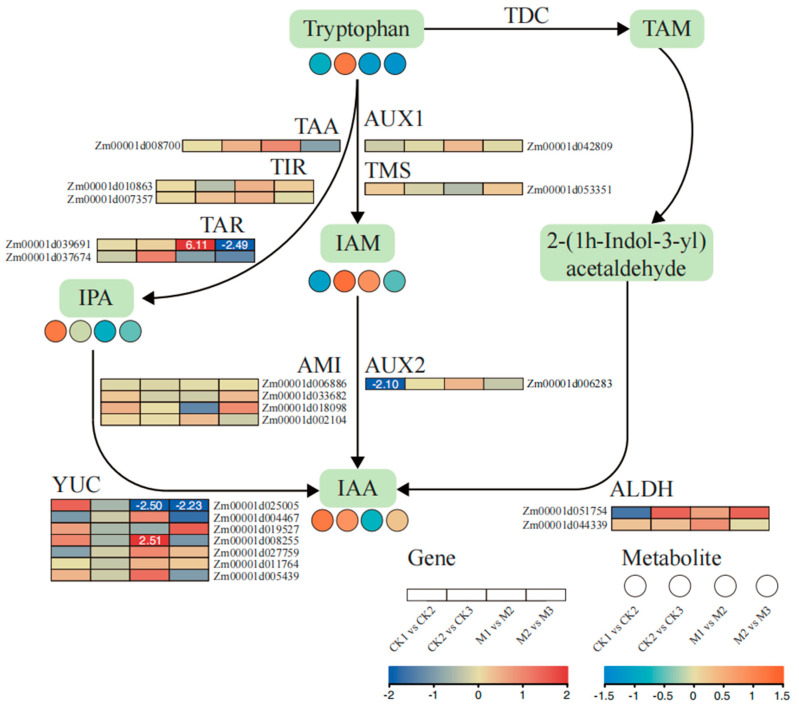
Pathways associated with indole−3−acetic acid (IAA) biosynthesis in maize. An expression heatmap of the key metabolites and transcripts for IAA is shown; red indicates up−regulation, blue indicates down−regulation. Enzyme and gene abbreviations: Tryptophan decarboxylase (TDC), Acetaldehyde dehydrogenase (ALDH), TRYPTOPHAN AMINOTRANSFERASE OF ARABIDOPSIS (TAA), Toll and Interleukin−1 receptor−like (TIR, TAR), YUCCA (YUC).

According to the KEGG enrichment analysis of DEGs and DAMs, phenylpropanoid biosynthesis, tryptophan metabolism, and plant hormone signal transduction were significantly enriched in each group. Therefore, the changes in genes and metabolites involved in auxin and Trp synthesis at the different growth stages were investigated (Figure 6 and Figure S6). The expression patterns of key enzymes and genes were analyzed, including anthranilate synthase (ASA), phosphoribosylanthranilate-transferase (PTA), phosphoribosylanthranilate isomerase (PAI), indole-3-glycerolphosphate synthase (IGS), tryptophan synthase (TSA and TSB), tryptophan decarboxylase (TDC), acetaldehyde dehydrogenase (ALDH), TRYPTOPHAN AMINOTRANSFERASE of ARABIDOPSIS (TAA), Toll and interleukin-1 receptor-like (TIR and TAR), and YUCCA (YUC). In auxin biosynthesis, five DEGs were identified based on the transcriptome data, including two *TAR* (Zm00001d039691 and Zm00001d037674), one *AMI* (Zm00001d018098), five *YUC* (Zm00001d025005, Zm00001d004467, Zm00001d019527, Zm00001d008255, and Zm00001d005439), one *AUX2* (Zm00001d006283), two *ALDH* (Zm00001d051754 and Zm00001d044339), and one *TAA* (Zm00001d008700). To verify the reliability of the transcriptome data, qRT-PCR was used to quantify the expression of these genes. The qRT-PCR results were consistent with the RNA-Seq data, which further verified the reliability of the transcriptome data (Figure S7).

In addition, the expression of key metabolites was analyzed. A total of 12 metabolites were identified in the auxin biosynthesis pathway and the Trp synthesis pathway (Figure 6 and Figure S6), comprising chorismate, anthranilate, 5-phosphoribosylanthranilate, 1-(O-carboxyphenylamino)-deoxyribulose-5-phosphate, indole-3-glycerol phosphate, indole, Trp, IPA, IAM, TAM, 2-(1H-indol-3-yl)acetaldehyde, and IAA. Similarly, the expression levels of the metabolites also changed. In general, the IPA pathway contained more DEGs, including Zm00001d039691, Zm00001d037674, Zm00001d025005, Zm00001d004467, Zm00001d019527, Zm00001d008255, and Zm00001d005439.

### 2.7. Weighted Gene Co-Expression Network Analysis

**Figure 7:**
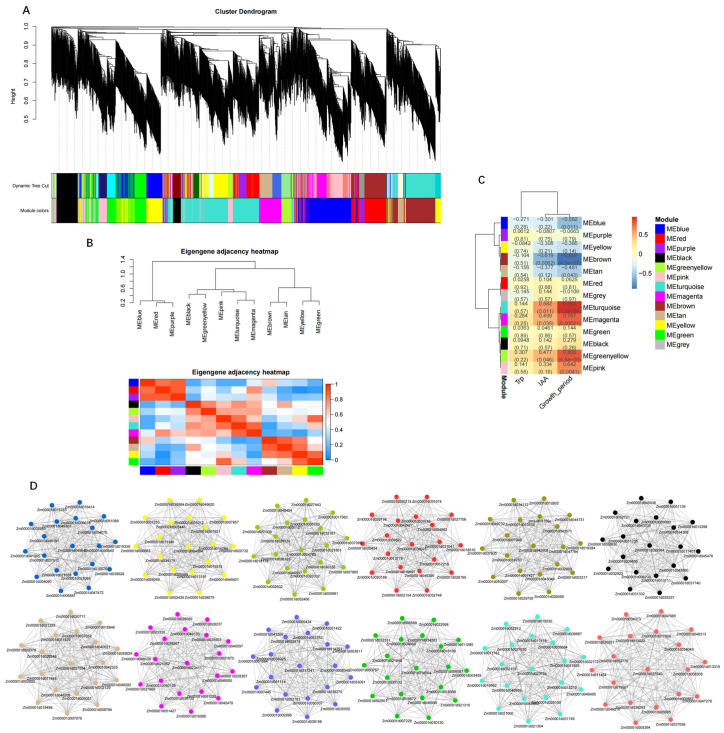
Weighted gene coexpression network for maize. (**A**) Cluster analysis dendrogram of differentially expressed genes (DEGs), with dissimilarity based on the topological overlap, together with assigned module colors. The clustered branches represent different modules, and each line represents one DEG. (**B**) Heatmap of connectivity of eigengenes. (**C**) Module–trait associations. Each row corresponds to a module characteristic gene (eigengene) and each column corresponds to a trait. Each cell contains a corresponding correlation coefficient and *p*-value. (**D**) Visualization of network relationships of hub genes in 12 modules.

Using the 4843 DEGs as the source data, a WGCNA analysis was conducted to analyze the relationship between genes. Under a soft threshold (β = 14), the scale-free network fit index was *R^2^* > 0.85 and the average connectivity approached 0 (Figure S8). Therefore, the soft threshold β = 14 was determined to construct a weighted co-expression network and 13 modules were resolved (Figure 7A). The number of genes in each module varied greatly, ranging from 419 genes in the turquoise module to 19 genes in the gray module. To detect interactions between these gene modules, a correlation thermogram analysis was performed on these modules (Figure 7B). The correlations among these modules were strong. Next, the correlations between the Growth_period, IAA and Trp contents, and the expression patterns of each module were analyzed (Figure 7C). Five modules (blue, green-yellow, pink, turquoise, and magenta) were strongly correlated with Growth_period, IAA, and Trp, whereas the other modules were weakly correlated with these traits.

Next, the changes in gene expression in these highly correlated modules and the relationship between the modules and traits were analyzed. This study analyzed the key gene modules and genes associated with auxin synthesis. To identify the crucial genes involved, 12 gene regulatory networks were constructed for the modules blue, red, purple, black, green-yellow, pink, turquoise, magenta, brown, tan, yellow, and green (Figure 7D) to visualize the association between genes. The hub genes were closely connected with each other and showed strong connectivity. It is worth noting that we identified genes involved in the auxin biosynthesis pathway among the 231 hub genes, especially *YUC* (Zm00001d025005, Zm00001d004467, Zm00001d019527, Zm00001d008255, and Zm00001d005439) from the turquoise module, *AUX2* (Zm00001d006283) from the green-yellow module, and *TAR* (Zm00001d039691) and *AMI* (Zm00001d018098) from the magenta module. These results revealed that the magenta, turquoise, and green-yellow modules played an important role in auxin biosynthesis and Growth_period, which further indicated that the *AUX2*, *YUC*, *TAR*, and *AMI* genes exerted significant effects on the growth period by affecting auxin synthesis.

## 3. Discussion

As an important food crop, research on the growth and development of maize is crucial to improve the yield per unit area. An increasing number of studies have investigated the auxin synthesis pathways of maize, but some of the mechanisms remain unclear [21]. The accumulation and regulatory mechanism of auxins in maize remain uncertain. In the present study, transcriptomic and metabonomic data were analyzed to understand the accumulation of auxins. By analyzing the accumulation of auxin metabolites at different growth stages, specific differences in the accumulation of auxin biosynthesis-related metabolites were detected.

Changes in the transcriptome and metabolome in maize leaves were analyzed at different growth stages. As expected, significant changes in auxin-related genes and metabolites at different growth stages were detected. The biosynthesis of Trp is a complex and important process. In particular, ASA, PAT, PAI, IGS, and TAS are catalyzed in the initial stage of auxin synthesis as the basis for subsequent stages [22,23]. Tryptophan is upstream of auxin synthesis, with chorismate first converted to Trp under the catalysis of ASA, PAT, PAI, IGS, and TAS [24,25]. Thus, Trp is a vital metabolite for auxin synthesis. Under the catalysis of different enzymes, Trp is the precursor for three branches: the first generates indole-3-acetaldoxime, the second synthesizes tryptamine, and the third produces indole-3-acetamide [26].

In addition, the present transcriptomic and metabonomic analyses revealed that genes encoding IGS and TSB were significantly correlated with downstream auxin precursors [27]. Both IGS and TSB play important roles in auxin synthesis as enzymes responsible for producing important auxin compounds [28]. The catalytic product of IGS, indole-3-glycerophosphate (InGP), is the first compound with an indole ring generated by the pathway. Previous experiments show that IGS may play a key role in the secondary metabolism of IAA and certain other indole derivatives [29,30]. Next, TSA catalyzes the conversion of InGP to indole [31,32], and TSB catalyzes the combination of indole and serine to form Trp to complete the final two steps of the Trp synthesis pathway catalyzed by tryptophan synthase [33]. The TSB1-deficient mutant Trp 2-1 accumulates IAA contents 50-fold higher than that of the wild type [34]. In the inbred maize line with a short growth period (the M group of this study), the expression of genes encoding *TSB* (Zm00001d049610, Zm00001d046676, and Zm00001d024702) tended to initially increase and then decrease, while the IAA content first decreased and then increased, which was consistent with the results of Wright et al. (1991) [34]. In addition, through a WGCNA analysis, it was found that many photopigment genes—CRY1, CRY2, PHYA, and PHYB—and photosensitive transcription factors—HY2, HY5, BBX, and PIF—had a high correlation with the auxin concentration at different developmental stages of maize. Xu et al. found that CRY1 and PHYB interacted with AUX/IAA, respectively, inhibiting the interaction of auxin receptor TIR1 with AUX/IAA, thereby inhibiting the degradation of auxin-induced AUX/IAA protein [35]. HY5 promotes auxin signal transduction by inhibiting the expression of the auxin negative regulatory gene IAA4 and IAA7, and the expression of IAA7 gene increases the auxin concentration against the background of hy5 abruption [36]. The wide range of light signals affects the biosynthesis process of auxin, which may be the reason for the large difference in auxin content between CK and M at the same growth stage.

Pollmann et al. (2006) cloned *AtAMI1* encoding indole-3-acetamide hydrolase in *Arabidopsis thaliana* and showed that the protein is localized in the cytoplasm [37]. In vitro experiments indicated that indole-3-acetamide significantly affected the conversion efficiency of Trp to IAA catalyzed by an *A. thaliana* extract protein, but had no significant effect on Trp, indole-3-acetonitrile, and indole acetaldehyde [38]. Pollmann et al. (2009) [39] verified the existence of indole-3-acetamide hydrolase in plants, which plays a role in the conversion of Trp to IAA. In the IPA pathway, Trp is converted to IPA through the activity of tryptophan amino-transferase (TAA) and the related proteins TAR1/TAR2 [40,41,42]. This process has been detected previously in maize and *Arabidopsis* [43,44]. In the present study, we observed that the expression level of a *TAR* gene (Zm00001d039691) in the M-group was unchanged during IAA synthesis, which was contrary to the trend in IAA content and differed from the results of previous research. However, a previous study has shown that the ethylene response inhibitor L-Kyu may suppress expression of the *TAR* gene and reduce auxin accumulation [45]. This warrants further study in maize.

Transcriptome analysis is an important method to monitor gene expression in organisms, and the metabolome is the basis and direct embodiment of the biological phenotype [46]. Transcriptome sequencing generates substantial information on DEGs and the regulators of metabolic pathways, but has difficulty in determining the key signaling pathways because the genes and phenotypes are weakly correlated with each other. Therefore, integration of metabonomic and transcriptomic data is an effective method to clarify the crucial genes and metabolites in a biosynthesis pathway. In the present study, the crucial genes and metabolites involved in the auxin biosynthesis of maize at different growth stages were analyzed using metabonomic and transcriptomic data. The results revealed that the gene expression and metabolite accumulation at different stages were significantly different from each other.

## 4. Materials and Methods

### 4.1. Plant Materials

Maize was cultivated and harvested in a greenhouse of Jilin Agricultural University (Longitude: 125.410385, Latitude: 43.810433). The plants were grown under a photoperiod of 8 h/16 h (light/dark). We kept the indoor temperature at 28 °C and the soil temperature at 24 °C, applied bottom fertilizer before sowing, and irrigated once every 10 days.

The inbred maize line with a long growth period was designated CK (selected and bred in the early stage of the laboratory; the growth period is 120 days and the plant height is 180 cm), and the inbred line with a short growth period was designated M (selected and bred in the early stage of the laboratory; the growth period is 90 days and the plant height is 150 m). It is planted in a small area with a row length of 5 m, row spacing of 30 cm, and plant spacing of 20 cm. Leaves were sampled from plants at the stages of jointing (1217-1, 1217-2, and 1217-3), tasseling (1222-1, 1222-2, and 1222-3), and pollen release (1231-1, 1231-2, and 1231-3). The codes in parentheses denote the date of collection and the individual sample; for example, 1217-1 is the first sample collected on 17 December. Some of the sampled materials were frozen in liquid nitrogen and stored at −80 °C for extraction of RNA and metabolites, whereas the remaining material was used for extraction of auxin and tryptophan (Trp). Three biological replicates were analyzed in all experiments of this study.

### 4.2. Determination of Auxin and Trp Contents

An Arc high-performance liquid chromatography system (Waters Corporation, Milford, MA, USA) was used for determinations. Leaf samples (0.1 g) at each sampling stage were ground to powder in liquid nitrogen with a pestle and mortar, and 6 mL of extract A (N-propanol:water:HCl = 2:1:0.002) was added. After shaking at 100 r·min^−1^ at 4 °C for 30 min, 3 mL of extract B (dichloromethane) was added and the solution was shaken at 100 r·min^−1^ at 4 °C for an additional 30 min, then centrifuged at 13,000 r·min^−1^ for 5 min at 4 °C. The organic layer solution was collected with a dropper, dried with nitrogen, and 1 mL of 50% methanol aqueous solution was added. After filtering through a 0.22-μm organic pinhole filter membrane, the filtrate was stored in a sample bottle at 4 °C for testing.

### 4.3. RNA-Sequencing and Differential Expression Gene Analysis in Maize

Total RNA was isolated from the leaf samples using a plant RNA Kit (CWBIO, Beijing, China). The cDNA libraries were sequenced on Illumina sequencing platforms (HiSeq™ 2500 and HiSeq X Ten). The filtered reads were mapped to the reference genome (Zea_mays; reference genome version: B73_RefGen_v4; source: http://plants.ensembl.org/Zea_mays/Info/Index, accessed on 2 March 2022) using HISAT2 software (http://ccb.jhu.edu/software/hisat2/index.shtml, accessed on 10 March 2022). The fragments per kilobase of exon per million mapped fragments (FPKM) values were calculated and used to evaluate gene expression. The DESeq2 R package (http://bioconductor.org/packages/stats/bioc/DESeq2/, accessed on 25 March 2022) was used to mine differentially expressed genes (DEGs) with the filtering criteria |log2(fold change)| > 1 and *p* < 0.05. To explore the functions and critical pathways of the DEGs, the Gene Ontology (GO; http://www.geneontology.org/, accessed on 7 April 2022) and Kyoto Encyclopedia of Genes and Genomes (KEGG; www.genome.jp/kegg, accessed on 27 April 2022) databases were used to determine the GO terms and pathways enriched among the DEGs.

### 4.4. Widely Targeted Metabonomic Analysis

We conducted a nontargeted metabonomic analysis to analyze the changes in metabolites of leaves at different growth stages. Metabolite analysis of the leaf samples was conducted by Shanghai Meiji Biomedical Technology Co., Ltd. (Shanghai, China). Principal component analysis (PCA) and orthogonal projections to latent structures–discriminant analysis (OPLS-DA) were separately used to analyze the differences in metabolites between samples. The Variable Importance in Projection (VIP version 1.6.2) of the OPLS-DA model was used to screen the differential metabolites. Metabolites with fold change ≥ 2 or fold change ≤ 0.5, and VIP ≥ 1 were considered to be differentially accumulated metabolites (DAMs). A comparison of metabolite accumulation at different growth stages was performed using the ropls R package (version 1.6.2) by performing PCA. The data were normalized, and all the samples were analyzed by means of cluster heatmaps.

### 4.5. Transcriptome and Metabolome Analysis

Based on the metabolite content and gene expression value in leaves at different growth stages, the DEGs and DAMs of auxin biosynthesis pathway in each control group were analyzed. First, the DEGs and DAMs associated with auxin biosynthesis were analyzed by pathway analysis. In addition, to explore the relationship between the transcriptome and metabolome, we mapped the DEGs and DAMs against the KEGG pathway database (http://www.genome.jp/kegg/, accessed on 28 April 2022) to obtain the pathway information common to both datasets.

### 4.6. Construction of Gene Co-Expression Network

To further study the genes significantly associated with Growth_period, and Auxin and Trp in maize leaves, a weighted gene co-expression network was generated using ‘WGCNA’ R package (https://horvath.genetics.ucla.edu/html/CoexpressionNetwork/Rpackages/WGCNA/, accessed on 7 May 2022). A topological overlap matrix was applied to reflect the co-expression relationship and the similarity between genes by calculating the adjacent matrix threshold on the matrix. A hierarchical clustering tree was constructed to visualize the similarity in expression among the DEGs. The strongly correlated genes were classified into co-expression modules and visualized as a gene network using Cytoscape (version 3.6.1). The 20 top-ranked genes with the strongest correlations were identified as the central genes in the co-expression network.

### 4.7. Real-Time Fluorescent Quantitative PCR Verification

Twelve DEGs were selected for verification by quantitative real-time PCR (qRT-PCR) analysis: Zm00001d039691, Zm00001d037674, Zm00001d025005, Zm00001d004467, Zm00001d019527, Zm00001d008255, Zm00001d005439, Zm00001d006283, Zm00001d051754, Zm00001d044339, Zm00001d008700, and Zm00001d018098. This method extracted 15 ng cDNA from the leaves of each growth stage. The experiment was performed with an Agilent Technologies Stratagene M×3000P, using SYBR pre-mixed Ex Taq following the manufacturer’s instructions. Actin was used as the internal reference gene. The relative expression level was calculated by the 2^−∆∆Ct^ method [47]. The primers used in this study are listed in Table S1.

## 5. Conclusions

This study has clarified auxin biosynthesis and its regulation in maize. The transcriptome of maize at three growth stages was analyzed and the crucial genes involved in auxin biosynthesis, namely *TSB* (Zm00001d046676 and Zm00001d049610), *IGS* (Zm00001d020008), *AUX2* (Zm00001d006283), *TAR* (Zm00001d039691), and *YUC* (Zm00001d025005 and Zm00001d008255), were identified. Potential MSTRG.11063 and MSTRG.35270 and MSTRG.21978 genes in auxin synthesis pathways were also obtained. Integrated analysis of transcriptome and metabolome data revealed that auxins accumulated more highly in leaves at the jointing stage but declined at the tasseling and pollen-release stages. As a result, the sensitivity of maize to auxins changes at different growth stages. The present results provide new insights for understanding the regulatory mechanism of auxin biosynthesis in maize.

## Data Availability

The datasets presented in this study can be found in the NCBI BioProject online repository (accession number: https://www.ncbi.nlm.nih.gov/bioproject/PRJNA788070, accessed on 14 February 2022).

## Supplementary Materials

The following supporting information can be downloaded at: https://www.mdpi.com/article/10.3390/ijms232113195/s1.

## References

[1] Fedoroff N.V. (1989). About maize transposable elements and development. Cell.

[2] Blázquez M.A., Nelson D.C., Weijers D. (2020). Evolution of Plant Hormone Response Pathways. Annu. Rev. Plant Biol..

[3] Gomes G., Scortecci K.C. (2021). Auxin and its role in plant development: Structure, signalling, regulation and response mechanisms. Plant Biol..

[4] Santner A., Calderon-Villalobos L.I., Estelle M. (2009). Plant hormones are versatile chemical regulators of plant growth. Nat. Chem. Biol..

[5] Dziewit K., Pěnčík A., Dobrzyńska K., Novák O., Szal B., Podgórska A. (2021). Spatiotemporal auxin distribution in Arabidopsis tissues is regulated by anabolic and catabolic reactions under long-term ammonium stress. BMC Plant Biol..

[6] Leyser O. (2018). Auxin Signaling. Plant Physiol..

[7] Kunkel B.N., Johnson J. (2021). Auxin Plays Multiple Roles during Plant-Pathogen Interactions. Cold Spring Harb. Perspect. Biol..

[8] Mano Y., Nemoto K. (2012). The pathway of auxin biosynthesis in plants. J. Exp. Bot..

[9] Korasick D.A., Enders T.A., Strader L.C. (2013). Auxin biosynthesis and storage forms. J. Exp. Bot..

[10] Yue K., Lingling L., Xie J., Coulter J.A., Luo Z. (2021). Synthesis and regulation of auxin and abscisic acid in maize. Plant Signal. Behav..

[11] Sugawara S., Hishiyama S., Jikumaru Y., Hanada A., Nishimura T., Koshiba T., Zhao Y., Kamiya Y., Kasahara H. (2009). Biochemical analyses of indole-3-acetaldoxime-dependent auxin biosynthesis in Arabidopsis. Proc. Natl. Acad. Sci. USA.

[12] Quittenden L.J., Davies N.W., Smith J.A., Molesworth P.P., Tivendale N.D., Ross J.J. (2009). Auxin biosynthesis in pea: Characterization of the tryptamine pathway. Plant Physiol..

[13] Sánchez-Parra B., Pérez-Alonso M.M., Ortiz-García P., Moya-Cuevas J., Hentrich M., Pollmann S. (2021). Accumulation of the Auxin Precursor Indole-3-Acetamide Curtails Growth through the Repression of Ribosome-Biogenesis and Development-Related Transcriptional Networks. Int. J. Mol. Sci..

[14] Lehmann T., Hoffmann M., Hentrich M., Pollmann S. (2010). Indole-3-acetamide-dependent auxin biosynthesis: A widely distributed way of indole-3-acetic acid production?. Eur. J. Cell Biol..

[15] Pérez-Alonso M.M., Ortiz-García P., Moya-Cuevas J., Lehmann T., Sánchez-Parra B., Björk R.G., Karim S., Amirjani M.R., Aronsson H., Wilkinson M.D. (2021). Endogenous indole-3-acetamide levels contribute to the crosstalk between auxin and abscisic acid, and trigger plant stress responses in Arabidopsis. J. Exp. Bot..

[16] Schütz A., Sandalova T., Ricagno S., Hübner G., König S., Schneider G. (2003). Crystal structure of thiamindiphosphate-dependent indolepyruvate decarboxylase from Enterobacter cloacae, an enzyme involved in the biosynthesis of the plant hormone indole-3-acetic acid. Eur. J. Biochem..

[17] Cao X., Yang H., Shang C., Ma S., Liu L., Cheng J. (2019). The Roles of Auxin Biosynthesis YUCCA Gene Family in Plants. Int. J. Mol. Sci..

[18] Qin M., Wang J., Zhang T., Hu X., Liu R., Gao T., Zhao S., Yuan Y., Zheng J., Wang Z. (2020). Genome-Wide Identification and Analysis on YUCCA Gene Family in Isatis indigotica Fort. and IiYUCCA6-1 Functional Exploration. Int. J. Mol. Sci..

[19] Uc-Chuc M.A., Pérez-Hernández C., Galaz-Ávalos R.M., Brito-Argaez L., Aguilar-Hernández V., Loyola-Vargas V.M. (2020). YUCCA-Mediated Biosynthesis of the Auxin IAA Is Required during the Somatic Embryogenic Induction Process in Coffea canephora. Int. J. Mol. Sci..

[20] Leontovyčová H., Trdá L., Dobrev P.I., Šašek V., Gay E., Balesdent M.H., Burketová L. (2020). Auxin biosynthesis in the phytopathogenic fungus Leptosphaeria maculans is associated with enhanced transcription of indole-3-pyruvate decarboxylase LmIPDC2 and tryptophan aminotransferase LmTAM1. Res. Microbiol..

[21] Lavy M., Estelle M. (2016). Mechanisms of auxin signaling. Development.

[22] Sadok I., Gamian A., Staniszewska M.M. (2017). Chromatographic analysis of tryptophan metabolites. J. Sep. Sci..

[23] Gruß H., Sewald N. (2020). Late-Stage Diversification of Tryptophan-Derived Biomolecules. Chem. A Eur. J..

[24] Chen M., Chen L., Zeng A.P. (2019). CRISPR/Cas9-facilitated engineering with growth-coupled and sensor-guided in vivo screening of enzyme variants for a more efficient chorismate pathway in E. coli. Metab. Eng. Commun..

[25] Xiu Z.L., Chang Z.Y., Zeng A.P. (2002). Nonlinear dynamics of regulation of bacterial trp operon: Model analysis of integrated effects of repression, feedback inhibition, and attenuation. Biotechnol. Prog..

[26] Woodward A.W., Bartel B. (2005). Auxin: Regulation, action, and interaction. Ann. Bot..

[27] Ouyang J., Shao X., Li J. (2000). Indole-3-glycerol phosphate, a branchpoint of indole-3-acetic acid biosynthesis from the tryptophan biosynthetic pathway in Arabidopsis thaliana. Plant J. Cell Mol. Biol..

[28] Jijón-Moreno S., Marcos-Jiménez C., Pedraza R.O., Ramírez-Mata A., de Salamone I.G., Fernández-Scavino A., Vásquez-Hernández C.A., Soto-Urzúa L., Baca B.E. (2015). The ipdC, hisC1 and hisC2 genes involved in indole-3-acetic production used as alternative phylogenetic markers in Azospirillum brasilense. Antonie Van Leeuwenhoek.

[29] Skirycz A., Reichelt M., Burow M., Birkemeyer C., Rolcik J., Kopka J., Zanor M.I., Gershenzon J., Strnad M., Szopa J. (2006). DOF transcription factor AtDof1.1 (OBP2) is part of a regulatory network controlling glucosinolate biosynthesis in Arabidopsis. Plant J. Cell Mol. Biol..

[30] Ferrer L., Mindt M., Suarez-Diez M., Jilg T., Zagorščak M., Lee J.H., Gruden K., Wendisch V.F., Cankar K. (2022). Fermentative Indole Production via Bacterial Tryptophan Synthase Alpha Subunit and Plant Indole-3-Glycerol Phosphate Lyase Enzymes. J. Agric. Food Chem..

[31] Li R., Jiang J., Jia S., Zhu X., Su H., Li J. (2020). Overexpressing broccoli tryptophan biosynthetic genes BoTSB1 and BoTSB2 promotes biosynthesis of IAA and indole glucosinolates. Physiol. Plant..

[32] O’Rourke K.F., D’Amico R.N., Sahu D., Boehr D.D. (2021). Distinct conformational dynamics and allosteric networks in alpha tryptophan synthase during active catalysis. Protein Sci. A Publ. Protein Soc..

[33] Abu-Zaitoon Y.M., Abu-Zaiton A., Tawaha A., Fandi K.G., Alnaimat S.M., Pati S., Almomani F.A. (2022). Evidence from Co-expression Analysis for the Involvement of Amidase and INS in the Tryptophan-Independent Pathway of IAA Synthesis in Arabidopsis. Appl. Biochem. Biotechnol..

[34] Wright A.D., Sampson M.B., Neuffer M.G., Michalczuk L., Slovin J.P., Cohen J.D. (1991). Indole-3-Acetic Acid Biosynthesis in the Mutant Maize orange pericarp, a Tryptophan Auxotroph. Science.

[35] Xu F., He S., Zhang J., Mao Z., Wang W., Li T., Yang H.Q. (2018). Photoactivated CRY1 and phyB Interact Directly with AUX/IAA Proteins to Inhibit Auxin Signaling in Arabidopsis. Mol. Plant.

[36] Jing Y., Lin R. (2020). Transcriptional regulatory network of the light signaling pathways. New Phytol..

[37] Pollmann S., Neu D., Lehmann T., Berkowitz O., Schäfer T., Weiler E.W. (2006). Subcellular localization and tissue specific expression of amidase 1 from Arabidopsis thaliana. Planta.

[38] Nemoto K., Hara M., Suzuki M., Seki H., Muranaka T., Mano Y. (2009). The NtAMI1 gene functions in cell division of tobacco BY-2 cells in the presence of indole-3-acetamide. FEBS Lett..

[39] Pollmann S., Düchting P., Weiler E.W. (2009). Tryptophan-dependent indole-3-acetic acid biosynthesis by ‘IAA-synthase’ proceeds via indole-3-acetamide. Phytochemistry.

[40] Suzuki M., Yamazaki C., Mitsui M., Kakei Y., Mitani Y., Nakamura A., Ishii T., Soeno K., Shimada Y. (2015). Transcriptional feedback regulation of YUCCA genes in response to auxin levels in Arabidopsis. Plant Cell Rep..

[41] Bagley M.C., Stepanova A.N., Ekelöf M., Alonso J.M., Muddiman D.C. (2020). Development of a relative quantification method for infrared matrix-assisted laser desorption electrospray ionization mass spectrometry imaging of Arabidopsis seedlings. Rapid Commun. Mass Spectrom..

[42] Hagen G. (2015). Auxin signal transduction. Essays Biochem..

[43] Kriechbaumer V., Park W.J., Gierl A., Glawischnig E. (2006). Auxin biosynthesis in maize. Plant Biol..

[44] Perez V.C., Dai R., Bai B., Tomiczek B., Askey B.C., Zhang Y., Rubin G.M., Ding Y., Grenning A., Block A.K. (2021). Aldoximes are precursors of auxins in Arabidopsis and maize. New Phytol..

[45] He W., Brumos J., Li H., Ji Y., Ke M., Gong X., Zeng Q., Li W., Zhang X., An F. (2011). A small-molecule screen identifies L-kynurenine as a competitive inhibitor of TAA1/TAR activity in ethylene-directed auxin biosynthesis and root growth in Arabidopsis. Plant Cell.

[46] Zhang A., Yang H., Ji S., Tian C., Chen N., Gong H., Li J. (2022). Metabolome and Transcriptome Analyses of Anthocyanin Accumulation Mechanisms Reveal Metabolite Variations and Key Candidate Genes Involved in the Pigmentation of Prunus tomentosa Thunb. Cherry Fruit. Front. Plant Sci..

[47] Livak K.J., Schmittgen T.D. (2001). Analysis of relative gene expression data using real-time quantitative PCR and the 2(-Delta Delta C(T)) Method. Methods.

