# Supplementary material for: The Integration of Metabolomics and Transcriptomics Provides New Insights for the Identification of Genes Key to Auxin Synthesis at Different Growth Stages of Maize
Source: Int J Mol Sci. 2022 Oct 30;23(21):13195. doi: 10.3390/ijms232113195 (PMC9659120; doi:10.3390/ijms232113195)
Supplement: Supplementary file 1 [file ijms-23-13195-s001.zip › Supplementary fig.pdf]

Integration of metabolomics and transcriptomics provides new insights for identification of key genes for auxin synthesis at different growth stages of maize

Supplementary

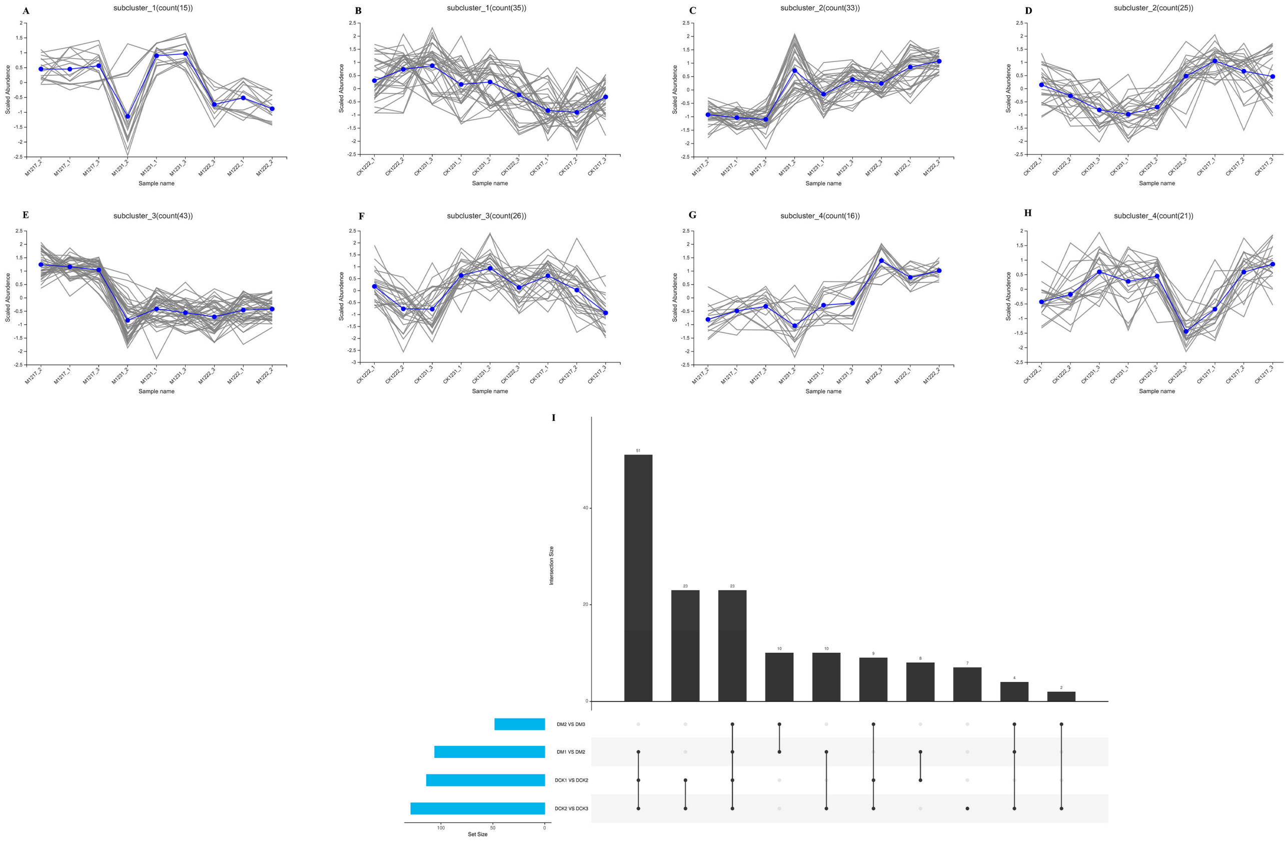

Figure S1: A–H: K-means analysis of differentially accumulated metabolites (DAMs). I: UpSet diagram of DAMs in different comparison groups.

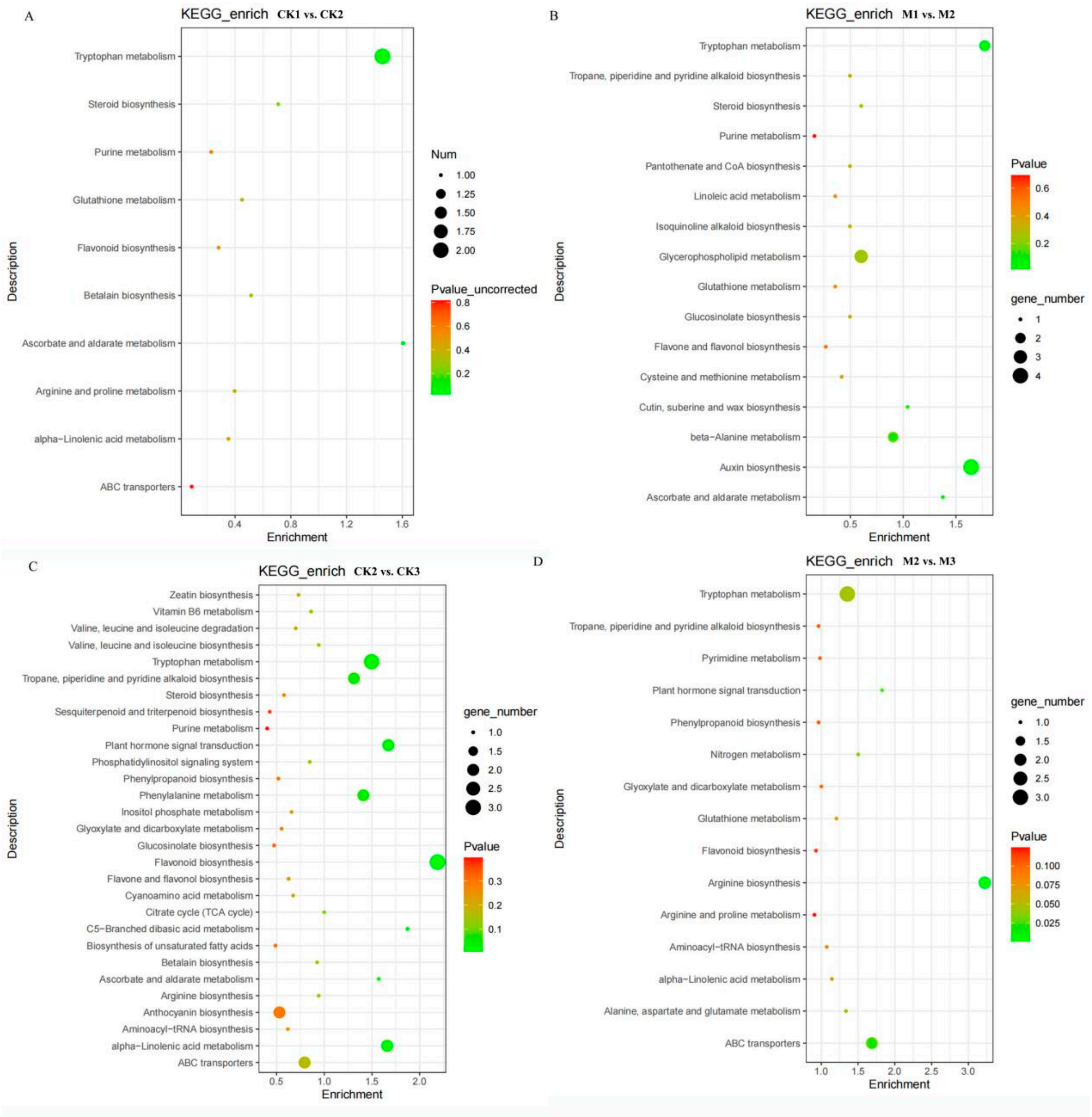

Figure S2: KEGG pathway enrichment bubble diagram. The Y-axis shows the name of the pathway and the X-axis represents the Rich factor. The larger the Rich factor, the greater the degree of enrichment. (A) CK1 vs. CK2, (B) M1 vs. M2, (C) CK2 vs. CK3, and (D) M2 vs. M3.

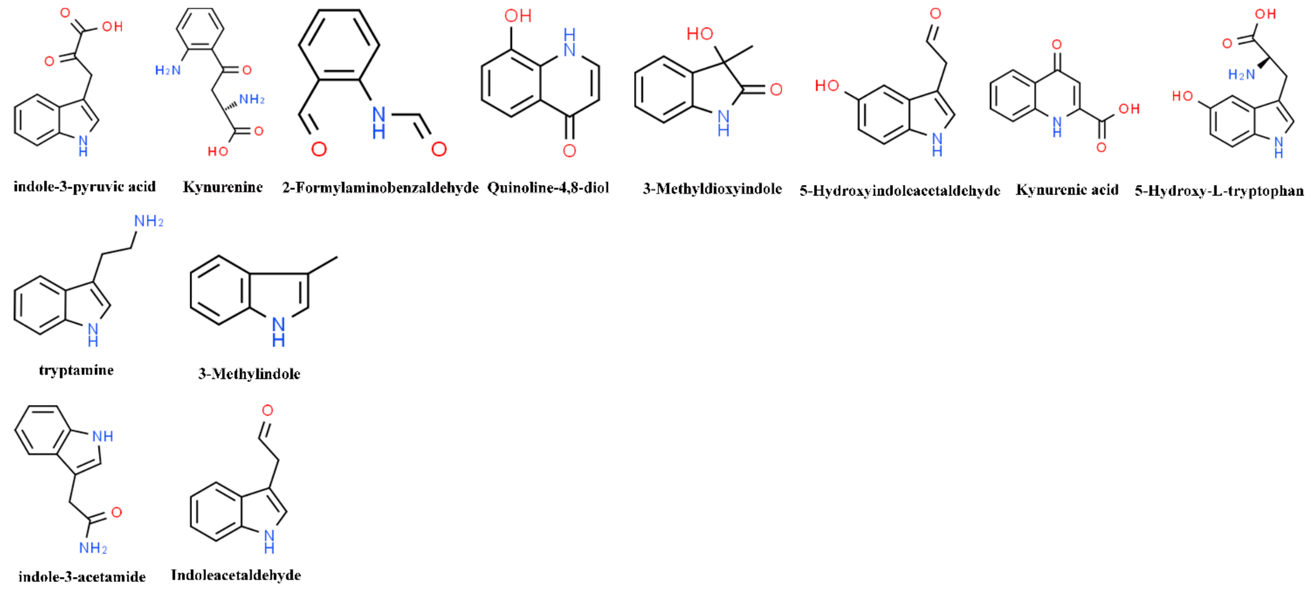

Figure S3: Metabolome screening yields metabolites structurally similar to IPA, IM, and TAM.

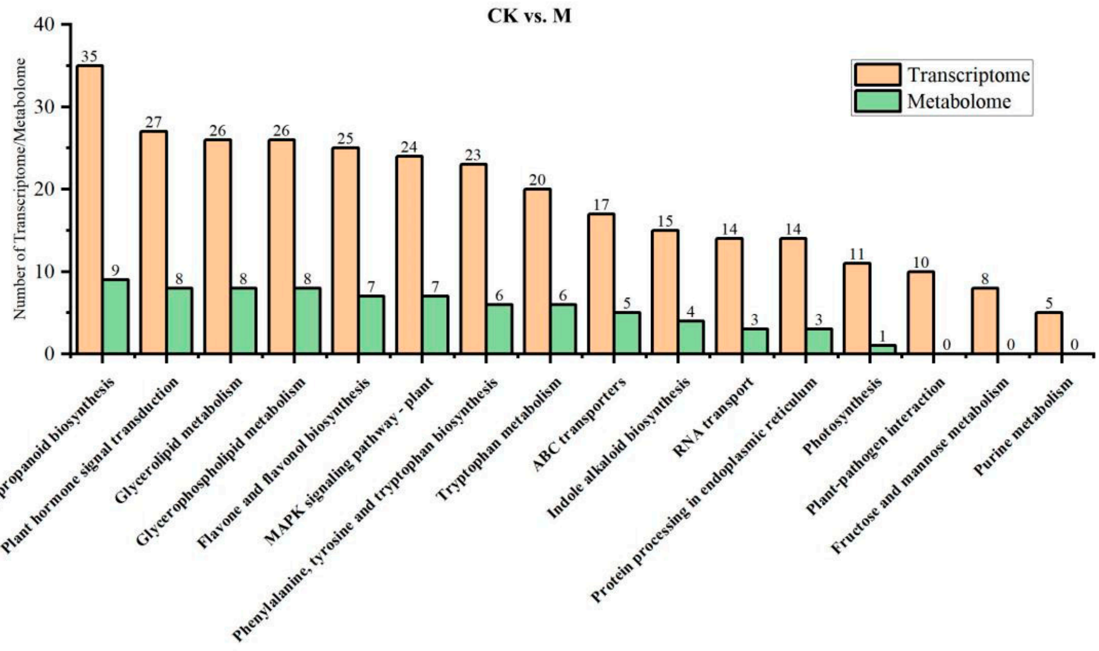

Figure S4: Integration of the ten most highly enriched KEGG pathways for differentially expressed genes and differentially accumulated metabolites.

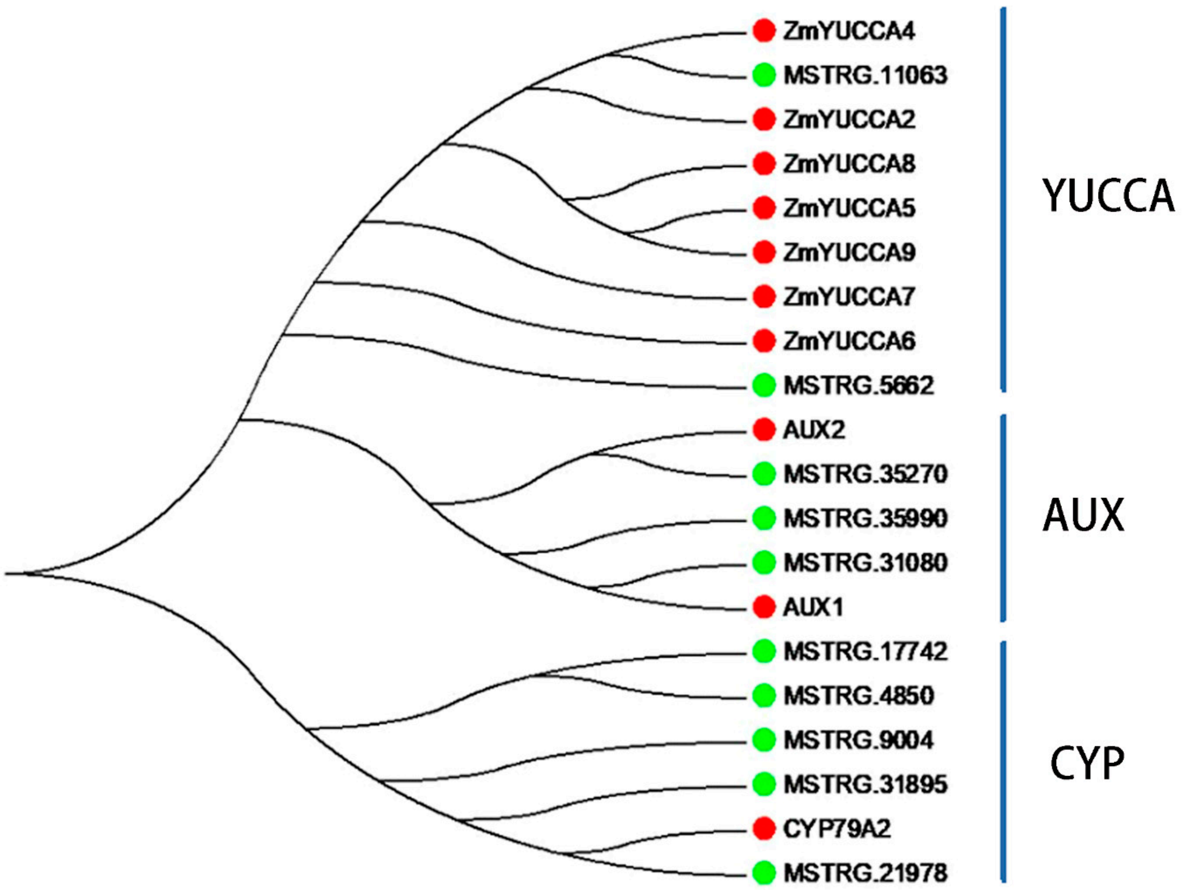

Figure S5: Evolutionary tree analysis of known and uncharacterized genes. Red is a known gene and green is a characterization gene.

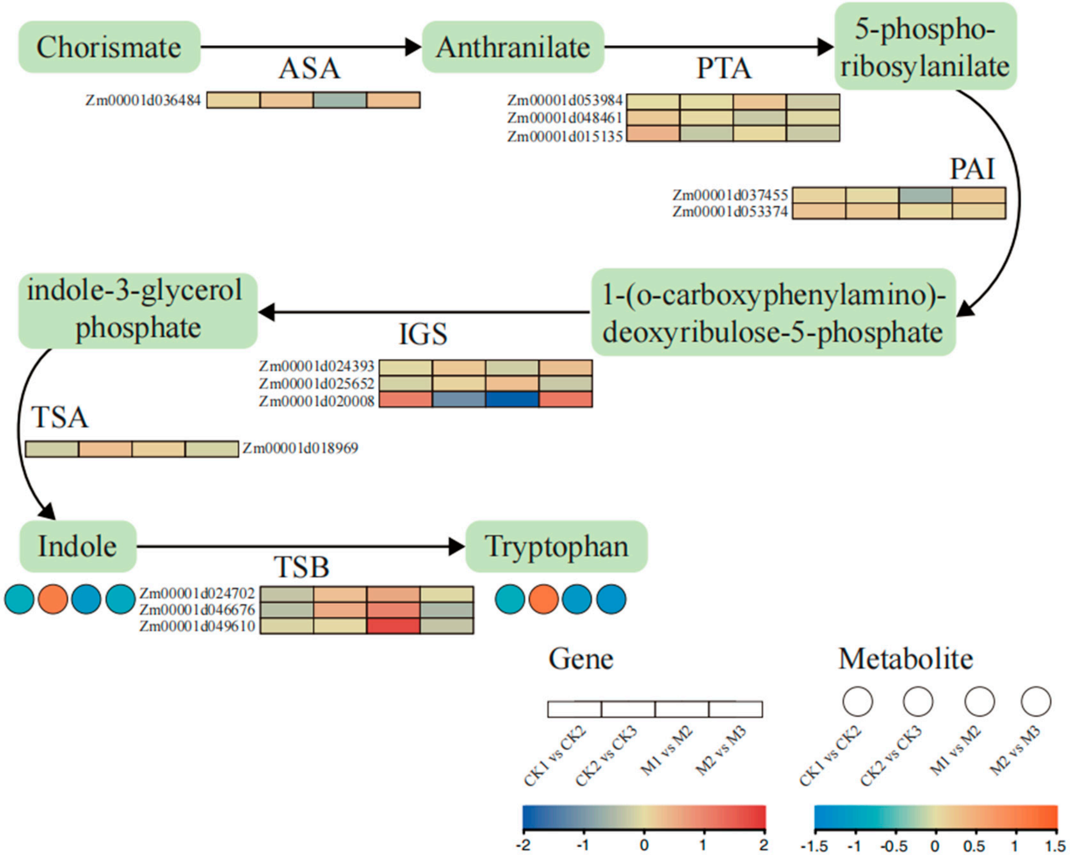

Figure S6: Pathways associated with tryptophan (Trp) biosynthesis in maize. An expression heatmap for the key metabolites and transcripts for Trp is shown; red indicates up-regulation, blue indicates down-regulation. Enzyme gene abbreviations: anthranilate synthase (ASA), phosphoribosylanthranilate transferase (PTA), phosphoribosylanthranilate isomerase (PAI), indole-3-glycerolphosphate synthase (IGS), tryptophan synthase (TSA and TSB).

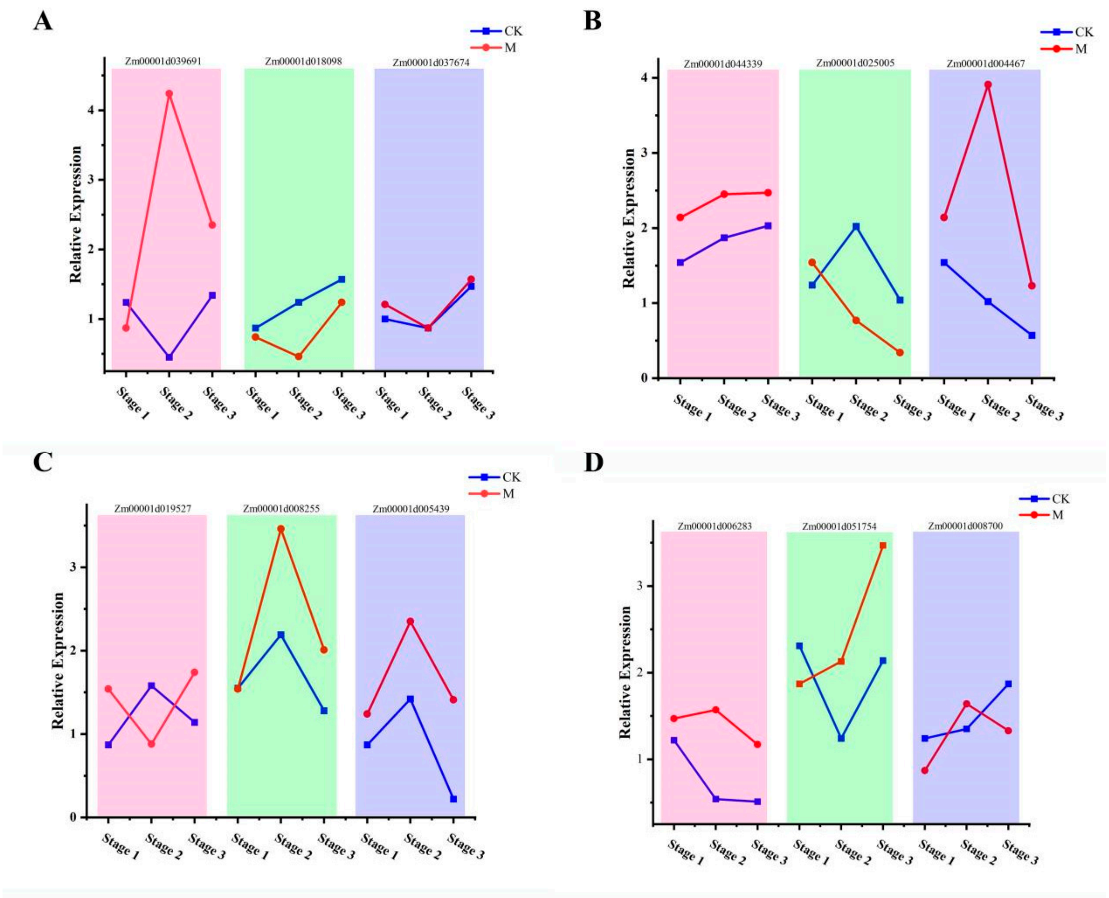

Figure S7: Analysis of the relative expression levels of 12 genes in the IAA biosynthesis pathway by qRT-PCR. Stage 1, jointing; Stage 2, tasseling; Stage 3, pollen dispersal.

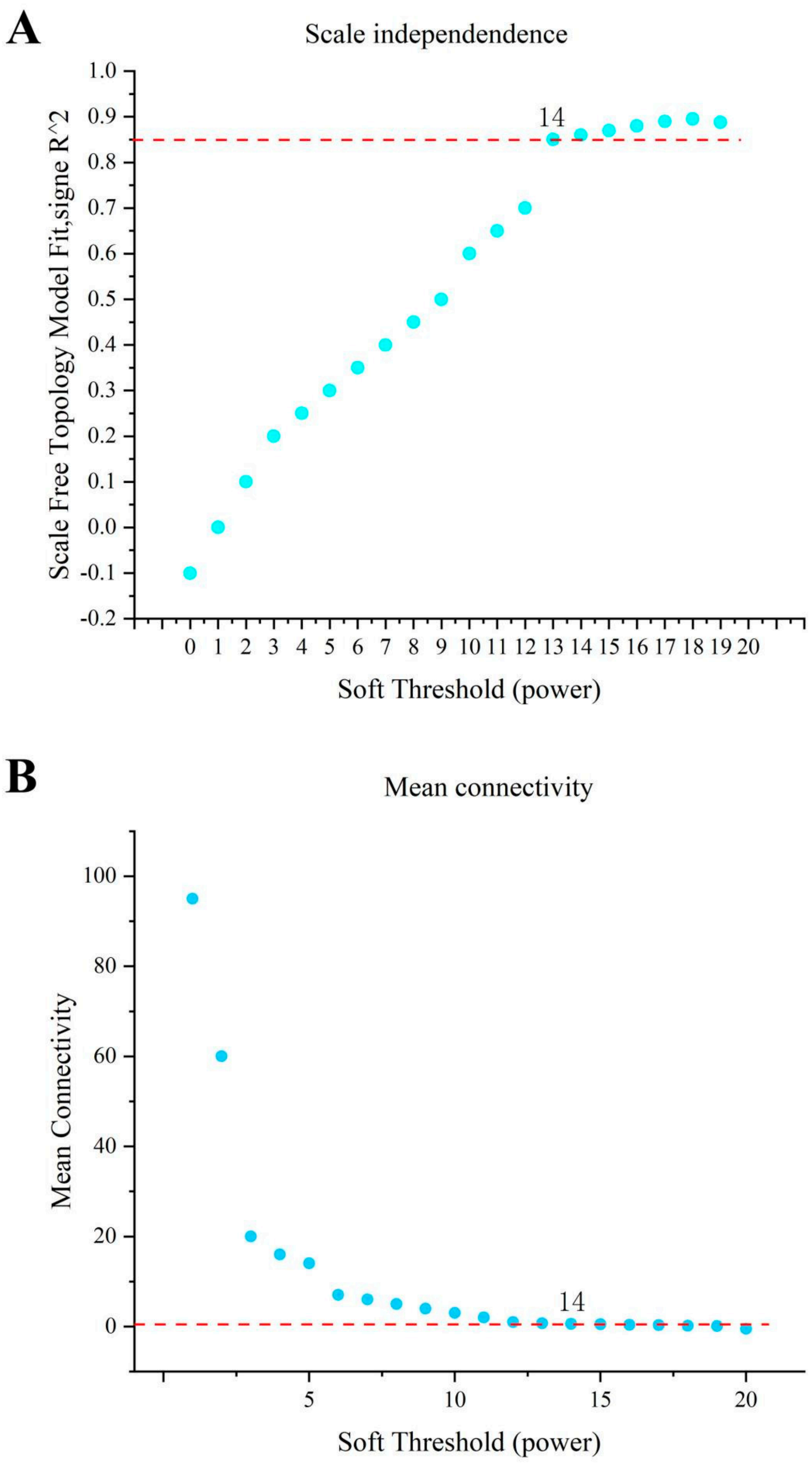

Figure S8: Determination of soft-thresholding power. (A) Analysis of the scale-free topology model fitting index. Red line is R2 = 0.85. (B) Mean connectivity for soft-thresholding power. Red line is β = 14.
