# Supplementary material for: The Integration of Metabolomics and Transcriptomics Provides New Insights for the Identification of Genes Key to Auxin Synthesis at Different Growth Stages of Maize
Source: Int J Mol Sci. 2022 Oct 30;23(21):13195. doi: 10.3390/ijms232113195 (PMC9659120; doi:10.3390/ijms232113195)
Supplement: Supplementary file 1 [file ijms-23-13195-s001.zip › Table S1.pdf]

Table S1 Genes IDs and primers used in the quantitative real-time PCR (qRT-PCR) experiments.

Primer_ID	Forward PCR Primer (5′-3′)	Reverse PCR Primer (5′-3′)
Actin	TCCCAAGGCAAACAGAGAAA	GGCCACTAGCATATAGGGAAAG
Zm00001d039691	CACGGAGATGGGCCTAGTTG	TGCCCTACTCATGCATGCTC
Zm00001d018098	ATCTGGAACTGGGCCGTTTT	TGCTTCAGACATGTCACCCG
Zm00001d037674	GGTACGTGCGTAACAACAGC	TCTGCGACTCCTTGCATACG
Zm00001d025005	GTGGCTCGAGGTAAATCCGT	GCCGTCTTGGGGTATCCTTC
Zm00001d004467	AGTCAAGAGCATTCCCTTCGT	GGGTACCCGTCCTTGTTGAA
Zm00001d019527	TCAAGAGGTTCTTCCGTGGC	CCACGCACTATGGGATGTGA
Zm00001d008255	GCATCATCTTCACGCGATGG	AAGTCTGACGCATCACCAGG
Zm00001d005439	CGTGGAATGGGAGAAGCAGA	CGTGGAGTTACCAGGTCGTC
Zm00001d006283	GAGCCACACATCAGATCGCT	AGTTGCAGCCTTGCAGGATT
Zm00001d051754	CGCTCGGGAGGAGATATTCG	TTATTGCCCTGCGTGACTGT
Zm00001d008700	TGAACGTCCACGTACGCAT	GATGTCGTAGGCATCGTCCC
Zm00001d044339	TGCCTTCCCTCAAGCAACAT	CGGGTGTTGTTCGCTTTCTG
